# Reprogrammed fecal and mucosa-associated intestinal microbiota and weakened mucus layer in intestinal goblet cell- specific Piezo1-deficient mice

**DOI:** 10.3389/fcimb.2022.1035386

**Published:** 2022-11-08

**Authors:** Ying Liu, Feifei Fang, Yilin Xiong, Jiandi Wu, Xueyan Li, Gangping Li, Tao Bai, Xiaohua Hou, Jun Song

**Affiliations:** Department of Gastroenterology, Union Hospital, Tongji Medical College, Huazhong University of Science and Technology, Wuhan, China

**Keywords:** goblet cells, Piezo1, mucus layer, fecal and mucosa-associated intestinal microbiota, mechanoreceptor, MUC2

## Abstract

Dysfunction of the mucus layer allows commensal and pathogenic microorganisms to reach the intestinal epithelium, thereby leading to infection and inflammation. This barrier is synthesized and secreted by host goblet cells. Many factors that influence the function of goblet cells (GCs) have been studied. However, how the microenvironment surrounding GCs influences the mucus layer and microbiota of the colon is unclear. To explore the effect of GC Piezo1 on the mucus layer and microbiota in the colon, we generated an intestinal epithelial Piezo1 conditional knockout mouse model. The fecal-associated microbiota (FAM) and mucosa-associated microbiota (MAM) of the two groups were characterized based on amplicon sequencing of the 16S rRNA gene. Our results showed that GC Piezo1^-/-^ mice developed decreased GC numbers, thinner mucus layer, and increased inflammatory cytokines (e.g., CXCL1, CXCL2, IL-6) on the 7th day. In addition, decreased Spdef and increased DOCK4 were discovered in KO mice. Meanwhile, the diversity and richness were increased in MAM and decreased in FAM in the GC Piezo1^-/-^ group compared with the GC Piezo1^+/+^ group. We also observed increased abundances of Firmicutes and decreased abundances of Verrucomicrobiota and Actinobacteriota in the MAM of the GC Piezo1^-/-^ group. Additionally, BugBase predicts that potentially pathogenic bacteria may have increased in the inner mucus layer, which is consistent with the higher abundance of Helicobacter hepaticus, Lactobacillus johnsonii, Escherichia-Shigella and Oscillospiraceae in MAM. These results further support the hypothesis that the role of Piezo1 in GCs is important for maintaining the function of the mucus layer and intestinal microbiota balance in the mouse colon.

## Introduction

The intestinal mucus layer has an essential role in protecting the intestine against mechanical, chemical, and biological attacks and it contributes to the maintenance of intestinal homeostasis (Johansson et al., 2013a; Paone and Cani, 2020)., The mucus layer in the colon can be divided into two major mucus layers: an inner attached dense mucus layer and an outer nonattached less dense layer. The inner colon layer is normally devoid of bacteria and acts as the first line of defense for the intestinal epithelium (Johansson et al., 2008; Johansson et al., 2011)., The outer mucus layer supplies attachment sites and nutrients for microorganisms. When the inner mucus layer fails, bacteria reach the epithelial surface and then activate the immune system and trigger inflammation (Deplancke and Gaskins, 2001; Johansson et al., 2013b; Johansson and Hansson, 2016a).,, Similarly, the intestinal microbiota also plays a fundamental role in human health and is a critical factor that regulates GI physiology and pathophysiology. Altered microbiota composition is associated with a range of inflammatory, autoimmune, metabolic, and tumor diseases, as well as a number of behavioral disorders (Lynch and Pedersen, 2016; Tilg et al., 2020)., It is known that fecal and mucosal-associated microbiota are different ecosystems with different microbial diversity and compositions as well as metabolic and immunological functions. Thus, the mucus layer not only acts as an important factor for protecting the host against microbial invaders but also contributes to the mutualism between the host and microbes (Sommer et al., 2017).

The goblet cells (GCs) of the gastrointestinal tract specialize in producing and secreting a number of important proteins (Allaire et al., 2018). Muc2, CLCA1, FCGBP, ZG16, and TFF3 are the major components of the intestinal mucus (Johansson et al., 2009; Erickson et al., 2016)., Mucin secretion from goblet cells involves several complex biological processes and is regulated by many factors (e.g., pathogens, pre/probiotics, diet, food additives or contaminants, and antibiotics) (Neutra et al., 1982; Plaisancié et al., 1998; Brownlee et al., 2003; Willemsen et al., 2003; McGuckin et al., 2011).

There are different forms of mechanical stimulation in the intestinal cavity, such as pilling stress produced by intestinal peristalsis and contraction and shear stress by the flow of intestinal contents (Mercado-Perez and Beyder, 2022). The Piezo1 protein is a mechanically sensitive nonselective ion channel that can detect increased membrane tension and then transduce external physical stimuli into electrochemical activity that influences cell behavior (Volkers et al., 2015; Wu et al., 2017)., Furthermore, our previous research proved that the Piezo1 protein functions as a primary mechanoreceptor in GCs and is essential for regulating mucin2 expression (Xu et al., 2021). However, how GC Piezo1 shapes the mucus layer and the organization of microbiota in the colon remains unknown.

To further explore the specific connection between GC Piezo1 and the mucus layer and intestinal microbiota, we generated an intestinal epithelial Piezo1 conditional knockout mouse model. We investigated the alterations in the mucus layer and the composition of the microbiota.

## Materials and methods

### Mouse models

MUC2-Cre recombinase transgenic C57BL/6 mice (MUC-Cre TG mice), and Piezo1^flox/flox^ C57BL/6 mice (both were purchased from Shanghai Model Organisms Center, Inc.) to cross and generate mice with MUC2 gene specifically deleted in intestinal MUC2^+^cell (MUC2-Cre^+^Piezo1^flox/flox^ mice). Administer tamoxifen *via* intraperitoneal injection (75mg/kg, 20mg/ml) once every 24 hours for a total of 5 consecutive days. Here is a 7-day waiting period after the final injection (using an ACUC approved injection procedure). Mice were raised in the Animal Experimental Center of Tongji Medical College. Mice were bred in-house and either co-housed with their littermates or separated according to genotype after weaning and healthy male animals were used for experiments when they were 8 weeks old. All mice were raised under specific pathogen-free conditions. All experimental procedures were approved by the Ethics Committee of Tongji Medical College.

### Fecal and mucosal samples collection

Both tamoxifen inducible CreERT2 mice and WT mice were observed dynamically for 7 days after the Piezo1 on GCs was knockout. All samples were collected at the end of the observation period. All mice were humanely euthanized by CO_2_ asphyxiation. Colons were sterilely removed with forceps, then the contents and mucosa were scraped from the distal colon tissue (1.0-2cm from the anus) respectively using sterile slides after the tissue was opened. All samples were stored at −80°C for sequencing.

### Histopathology

All tissue was collected 1.5-2cm from the anus to represent the distal colon. The fixed colon tissues were embedded in paraffin, sectioned, and stained with hematoxylin and eosin for microscopic examination. The histological analysis was calculated based on inflammation severity, inflammation extent, and crypt damage as previously reported (Tang et al., 2021).

### Quantitative real-time PCR

Quantitative real-time reverse transcription RT−PCR (RT−PCR) was carried out in a LightCycler 480 using SYBR Green Transcription Master Mix (Roche Diagnostics, IN, USA) as previously described. Transcripts of Piezo1, Mucin2, TFF3, AGR2, and proinflammatory cytokines were amplified using specific primer pairs and conditions, while GAPDH was used as an internal control. Relative quantification of those genes was performed by the 2−ΔΔCtmethod. The primers are as follows:

GAPDH: TGAAGCAGGCATCTGAGG; CGAAGGTGGAAGAGTGGGAG. Mucin2: GCTGACGAGTGGTTGGTGAATG; GATGAGGTGGCAGACAGGAGAC. TFF3: TTGCTGGGTCCTCTGGGATAG; TACACTGCTCCGATGTGACAG.

AGR2: ACGAATGCCCACACAGTCAA; GCGTAGAGCCGGTTTGAGTA.

IL-6: AGGATACCACTCCCAACAGACCT; CAAGTGCATCATCGTTGTTCATAC.

TNF-α: CATCTTCTCAAAATTCGAGTGACAA; TGGGAGTAGACAAGGTACAACCC.

IL-1β: CCGTGGACCTTCCAGGATGA; GGGAACGTCACACACCAGCA.

IFN-γ: CAGCAACAGCAAGGCGAAA; TTGAATGCTTGGCGCTGGAC.

CXCL1: GCTGGGATTCACCTCAAGAA; TGGGGACACCTTTTAGCATC.

CXCL2: CCCTGGTTCAGAAAATCATCCA; GCTCCTCCTTTCCAGGTCAGT.

DOCK4: ACGGCTGGTACAGAGGATTTG; GCTGTTTCCACATGGTTCCC.

Hes1: TCAACACGACACCGGACAAAC; ATGCCGGGAGCTATCTTTCTT.

Spdef: AGAGCCCAAGGTCAGGGAGG;TGGACTGCCTGTGGCCTTTG.

Reg3b: TAGACCGTGCTTTCTGTGGC; TTCGGGATGTTTGCTGTCTGA.

Klf4-F: AGGCACACCTGCGAACTCA; CAGCCGTCCCAGTCACAGT.

Reg3g: ACGAATCCTTCCTCTTCCTCAG; GTCTTCACATTTGGGATCTTGC.

Ang4: TAGACTCGTCCCCAGTTGGA; CTGAGCCAGAGTTGGAGGAA.

### Alcian blue staining

The mice were killed after isoflurane anesthesia, and the colon was separated and fixed with Carnoy’s fluid at room temperature (Puchtler et al., 1970). After the tissue was embedded and sliced, the staining was carried out with Alcian Blue Periodic acid Schiff Kit (AB-PAS Kit) (Baso, China), and bright field images were captured. Next, a researcher who was unaware of the grouping measured the thickness as previously described (Erickson et al., 2015) (10 measurements per section/two sections per animal/five animals per group) with ImageJ.

### Immunofluorescence and immunohistochemistry

Immunofluorescence was carried out as previously described (Tang et al., 2021). Anti-Piezo1 antibody (1:200, Proteintech, 15939-1-AP), anti-AGR2 antibody (1:200, R&D Systems, AF6068) anti-muc2 antibody (1:200, Genetex, GTX100664) and were diluted at a ratio of 1 to 400 for staining. Alexa Fluor 488- and Alexa Fluor 594-conjugated secondary antibodies (1:200; Antgene, ANT024S). Nuclei were mounted with 4’,6’-diamidino-2-phenylindole (DAPI, 1:1000; Antgene, ANT063).

Immunohistochemistry (IHC) of colon tissues was performed using a VECTASTAIN Elite ABC kit and a DAB Detection kit (Boster Biological Technology Co., Ltd) following the manufacturer’s instructions with an anti-F4/80 antibody (1:200, Proteintech, 15939-1-AP).

### High-throughput sequencing and bioinformatics analysis

Ten fecal and mucosal samples (i.e., five from GC Piezo1**-/-** mice and five from GC Piezo1**+/+** mice) were randomly collected and homogenized with a bead-based technique on a FastPrep-24. Total bacterial DNA from the fecal samples was extracted using a FastPrep-24 system (MP Biomedicals, Santa Ana, CA, USA) and QIAamp DNA Stool Mini Kit (Qiagen, Hilden, Germany) and evaluated by 1% (w/v) agarose gel electrophoresis. PCR was performed with the following universal 16S rRNA primers (V3–V5 region). Both primers were linked to an Illumina sequencing adapter, and the reverse primer contained a sample barcode. PCR products were purified, and the concentrations were adjusted for sequencing on an Illumina MiSeq PE300 system (MajorBio Co., Ltd., Shanghai, China).

The data were analyzed on the Majorbio Cloud Platform (www.majorbio.com). Flash software (https://ccb.jhu.edu/software/FLASH/index.shtml) was used for the assemblage of contiguous sequences and removal if found to be too short after trimming for a base quality below 20. The Uparse method (http://drive5.com/uparse/) was used to cluster the contigs and remove chimeras using version 7 of usearch (http://www.drive5.com/usearch/). The optimized sequences were clustered into operational taxonomic units (OTUs) with 97% similarity and aligned using the Silva138/16s_bacteria database (http://www.arb-silva.de). In total, 649820 sequences were generated from 20 samples. Among them, 2241436 sequences were considered to be high-quality sequences with an average length of 418 bp per sequence. The total number of OTUs at the 97% similarity level was 1280. The minimum number of reads (30752 reads) subsample was taken from each sample for subsequent analysis. Alpha diversity analysis was performed using the Mothur software package (https://www.mothur.org/wiki/Download_mothur). Bray–Curtis similarities were used to construct a cluster dendrogram. Unweighted UniFrac distance metric analysis was conducted using OTUs from each sample, and principal component analysis in terms of the matrix of distance was performed. A metagenomic biomarker discovery approach was employed with LEfSe (linear discriminant analysis (LDA) coupled with effect size measurement), which performed a nonparametric Wilcoxon sum-rank test followed by LDA analysis using online software (http://huttenhower.sph.harvard.edu/galaxy/) to assess the effect size of each differentially abundant taxon. BugBase (https://www.mothur.org/wiki/Download_mothur) was used for the predictions of the functional profile of a microbial community.

### Statistical analysis

The results were expressed as mean ± mean standard error (SEM). Statistical analysis and graphical illustrations were performed using GraphPad PRISM 8.0.2 (GraphPad Software) in individual experiments. Mann–Whitney test was used for statistical analyses. * p<0.05, ** p<0.01, *** p<0.0001, ns = nonsignificant.

## Results

### Tendency toward spontaneous inflammation in GC Piezo1^-/-^ mice

**Figure 1:**
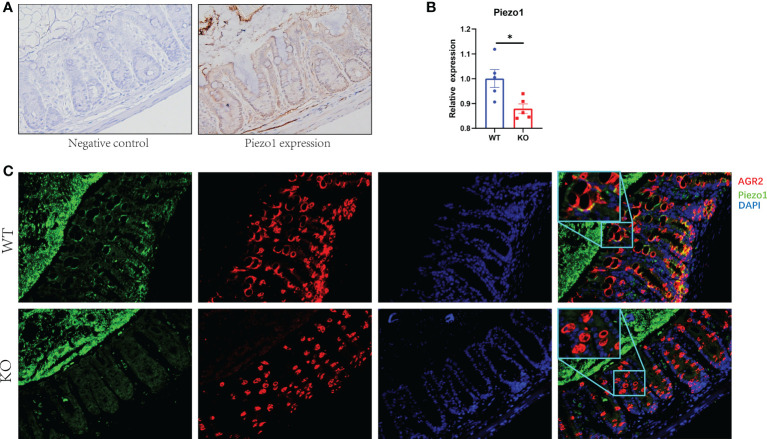
Expression of Piezo1 in colon. **(A)** Expression of Piezo1 in colon. **(B)** Relative mRNA expression of Piezo1. **(C)** The co-localization of AGR2 and GC Piezo1 in colon (400×). Data are expressed as the mean ± SEM (n =4-5). *p < 0.05.

**Figure 2:**
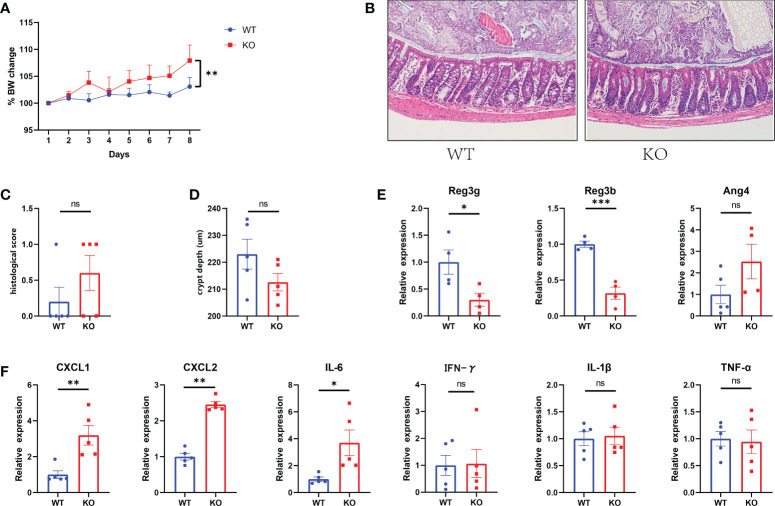
Tendency toward spontaneous inflammation in GC Piezo1^-/-^ mice. **(A)** The rate of body weight change. **(B)** Representative H&E staining images of colon tissue. 200×. **(C)** Histological scores of colons. **(D)** Colonic crypt depth was determined in sections with H&E staining. **(E)** The relative mRNA level of Ang4, Reg3b and Reg3g in colonic tissue. **(F)** The relative mRNA levels of CXCL1, CXCL2, IL-6, IFN-γ, TNF-α and IL-1β in colonic tissue. Data are expressed as the mean ± SEM. n =4-5. *p < 0.05, **p < 0.01, ***p < 0.001. ns, no significance.

Piezo1 was widely expressed in colonic tissue (**Figure 1A**). To evaluate the function of GC Piezo1 in the colon, we observed the mice for 7 consecutive days and validated the expression of GC Piezo1 was significantly decreased in GC Piezo1**-/-** mice, the expression of Piezo1 mRNA declined (**Figure 1B**). Piezo1 was mainly expressed on the GCs membrane (**Figure 1C**). Unexpectedly, the rate of body weight change was slower in WT mice (**Figure 2A**). But there was no significant change in structure, histological scores, or crypt depth between the two groups (**Figures 2B, D**). Notably, upregulated expression of the proinflammatory cytokine CXCL1, CXCL2, and IL-6 was observed in GC Piezo1**-/-** mice, although the expression levels of IFN-γ, TNF-α, and IL-1β were not significantly changed. But we investigated the expression levels of bactericidal peptides Reg3b and Reg3g were significantly downregulated and the expression of Ang4 was slightly increased (**Figures 2E, F**). These results suggested that GC Piezo1**-/-** mice might have a tendency toward spontaneous inflammation over a long period.

### Decreased GC numbers and weakened mucus layer in GC Piezo1-/- mice

**Figure 3:**
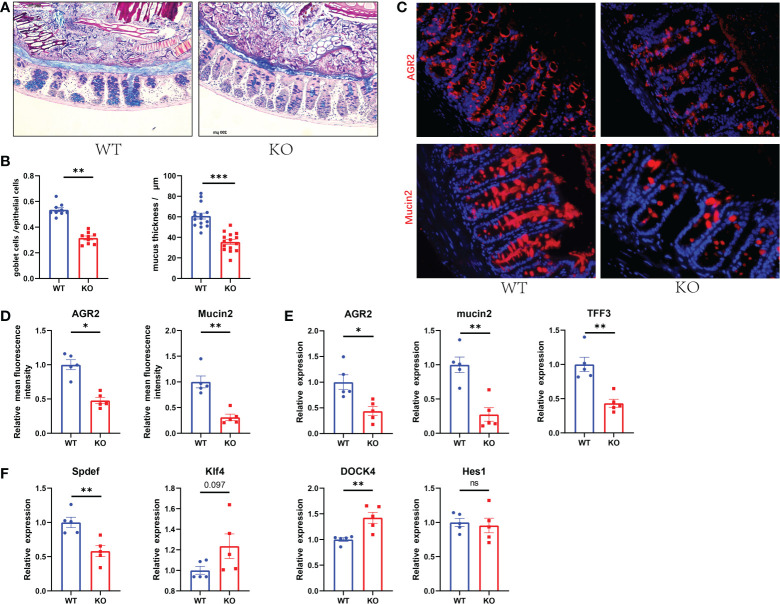
Decreased GC numbers and weakened mucus layer in GC Piezo1-/- mice. **(A)** The mucus layer in colon tissue by AB-PAS staining. 200×. **(B)** the number of goblet cells and the thickness of the inner mucus layer from two groups. **(C)** The typical immunofluorescent images of colon tissues were stained with DAPI (blue) and Mucin2 (red) or AGR2 (red) and were observed using a confocal laser-scanning microscope. 400×. **(D)** The relative mean fluorescence intensity of AGR2, Mucin2. **(E)** The relative mRNA expression of Mucin2, AGR2, and TFF3 in colonic tissue. **(F)** The relative mRNA expression of DOCK4, Hes1, Spdef, and Klf4 in colonic tissue. Data are expressed as the mean ± SEM. (n = 5). *p < 0.05, **p < 0.01, ***p < 0.001;. ns, no significance.

To determine the association between GC Piezo1 and mucus layer changes in the colon, we quantified the changed function of GCs and the mucus layer. We found that the expression of Mucin2 and AGR2, the thickness of the mucus, and the number of GCs in the colon were significantly decreased in GC Piezo1^-/-^ mice (**Figures 3A–E**). Of note, the changed distribution of AGR2 might result from the decreased protein production of Mucin2, TFF3 and AGR2 and the rearrangement of the cellular cytoskeleton. DOCK4 and Spdef, which served as critical regulators of goblet cell differentiation and Mucin2 production in the intestine, were observably changed, but Hes1 and Klf4 didn’t change. (**Figure 3F**). These results indicated that Piezo1 deficiency in GCs led to a thinner and weaker mucus layer, and the phenomenon might result from the dysfunction of goblet cell differentiation.

### The α-diversity and β-diversity indices among the 2 groups in the colon

**Figure 4:**
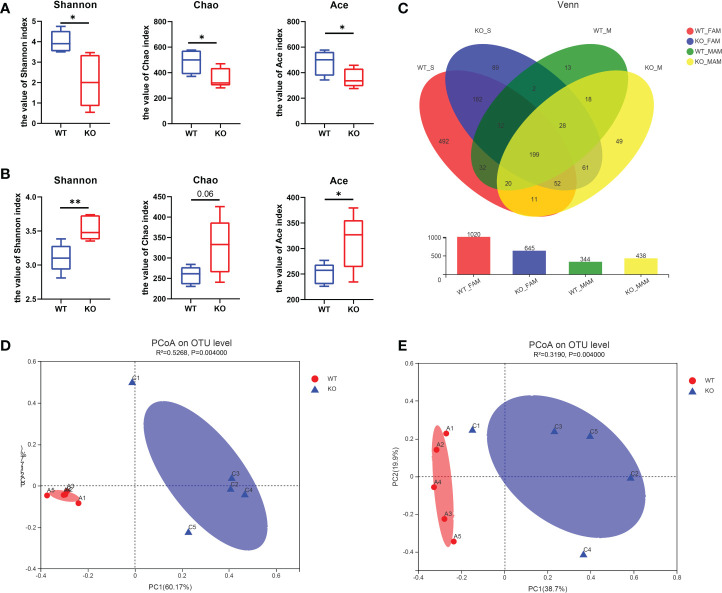
Characteristics of the alpha diversity in the two groups of mice. **(A, B)** Simpson index, Shannon index, Chao1 index, Ace index and Sobs index of FAM **(A)** and MAM **(B)** in each group (n=5). **(C)** Venn diagram showing exclusive features per group. **(D, E)** Plots of unweighted UniFrac principal coordinates were scored on the relative abundance of OTUs (97% similarity level) in the WT and GC Piezo1-/- groups. Each dot denotes a sample. Red dots represent the WT group, and blue dots represent the GC Piezo1-/- group. Differences were assessed by the Mann−Whitney-Wilcoxon test and denoted as follows: *, P < 0.05; **, P < 0.01; OTU, operational taxonomic unit; FAM, fecal-associated microbiota; MAM, mucosa-associated microbiota.

16S rRNA sequencing was performed to explore the exact changes in the microbiota in GC Piezo1**-/-** mice. Alpha-diversity analysis is mainly used to assess community diversity and richness. Among several α-diversity indices, the Shannon, Chao1, and Ace indices were significantly lower in the GC Piezo1**-/-** group in the FAM composition (P<0.05) (**Figure 4A**), while the Shannon and Ace indices were obviously higher in the GC Piezo1**-/-** group in the MAM composition (P<0.05) (**Figure 4B**) than in the GC Piezo1+/+ group. This inconsistent change between FAM and MAM suggested that Piezo1 deficiency in GCs could increase the diversity and abundance of MAM while reducing them in FAM.

The Venn diagrams showed that the OTUs were increased in MAM (344 vs. 438) but decreased in FAM (1020 vs. 645) in the GC Piezo1-/- group (**Figure 4C**), which means that Piezo1 on GCs affects the community structure of the gut microbiota. PCoA based on weighted UniFrac distances was utilized to measure the b-diversity. Adonis analysis revealed that both the FAM and MAM communities were significantly separated, with FAM (R2 = 0.5268; P = 0.0010) and MAM (R2 = 0.3190; P = 0.0010) (**Figures 4D, E**).

### Altered microbiota composition in GC Piezo1-/- mice

**Figure 5:**
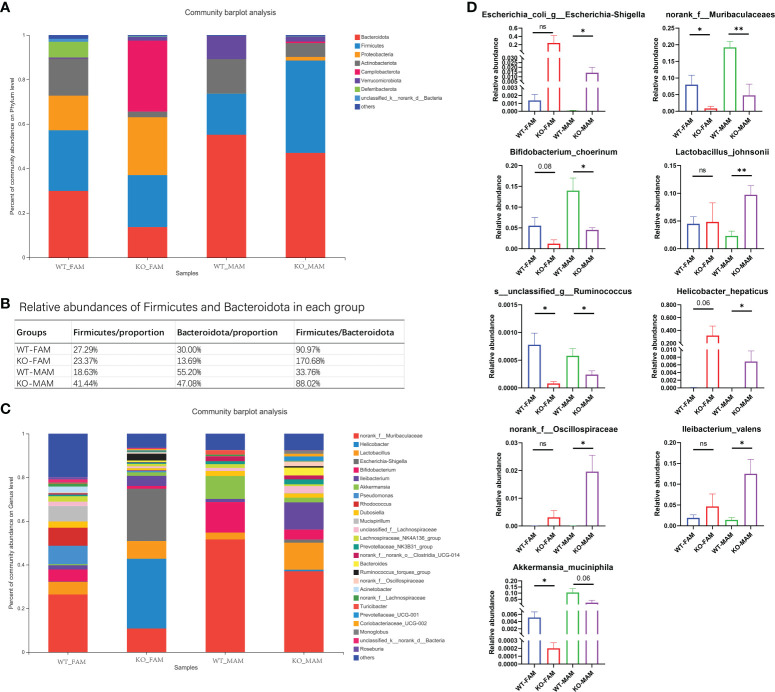
Bacterial composition of the different communities. Relative abundances of the gut microbiota at the phylum level **(A)** and genus level **(C)**; **(B)** proportion of Firmicutes and Bacteroidia in each group. **(D)** Relative abundances of nine kinds of bacteria in each group. FAM, fecal-associated microbiota; MAM, mucosa-associated microbiota. *p < 0.05, **p < 0.01, ns, no significance.

At the FAM phylum level, the GC Piezo1-/- group exhibited a significantly higher abundance of Campilobacterota phylum and a lower abundance of Actinobacteriota and Deferribacterota phyla than the GC Piezo1+/+ group. For MAM, Firmicutes were more abundant in the GC Piezo1-/- group, while the abundance of Verrucomicrobiota and Actinobacteriota was largely decreased in the GC Piezo1-/- group. At the FAM genus level, we observed a higher abundance of Helicobacter and Escherichia-Shigella and a lower abundance of norank_f_Muribaculaceae, Mucispirillum, Bifidobacterium, and Rhodococcus in the Piezo1-/- group than in the Piezo1+/+ group (**Figure 5A**). In addition, the ratio of Firmicutes/Bacteroidota (F/B) was markedly increased both in the FAM and MAM of the GC Piezo1-/- group (**Figure 5B**). At the MAM genus level, there was a higher abundance of Lleibacterium, Lactobacillus_johnsonii, Escherichia-Shigella, and a lower abundance of norank_f_Muribaculaceae, Bifidobacterium_choerinum, Akkermansia_muciniphila. Furthermore, we found that mucin-degrading bacteria such as Akkermansia muciniphila and Oscillospiraceae were decreased in GC Piezo1-/- group MAM (**Figure 5C**), which indicated that the thinner mucus was more likely caused by dysfunction due to Piezo1 deficiency in GCs than mucus degradation. In other words, mechanical stress is essential for maintaining the normal function of GCs.

Interestingly, bacteria such as Bifidobacterium, Muribaculaceae, Akkermansia muciniphila, and Lactobacillus johnsonii, which are beneficial for the body, were lower in both the FAM and MAM of the GC Piezo1-/- group. *Helicobacter hepaticus*, Ruminococcus, Escherichia- Shigella, and Oscillospiraceae, which are known as pathogenic bacteria, were increased in the MAM of the GC Piezo1-/- group (**Figure 5D**). This increased harmful bacteria in MAM demonstrated dysfunction of the mucus layer.

**Figure 6:**
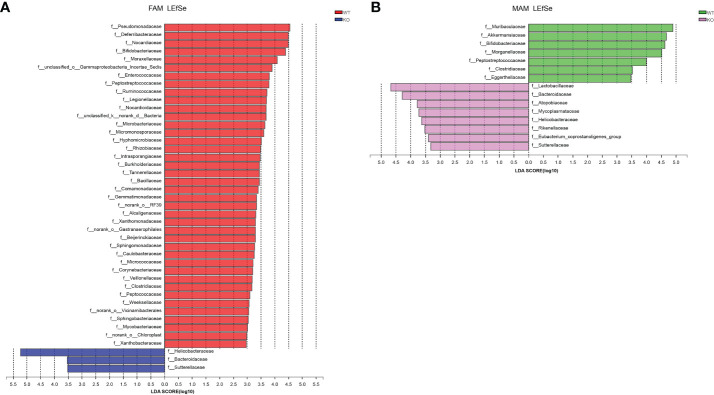
Bacterial composition of the different communities in the two groups. **(A, B)** Key microbiota contributing to the composition of gut microbiota in the fecal samples of the two groups in FAM **(A)** and MAM **(B)**; LDA, linear discriminant analysis.

To investigate the cladogram representation and the characteristic bacteria, LEfSe analysis was performed within the GC Piezo1**+/+** and GC Piezo1-/- mice. The differences in taxa between the two groups were detected by linear discriminant analysis (LDA). In GC Piezo1**-/-** mice, FAM, Helicobacteraceae, Campylobacteria, Proteobacteria, Gammaproteobacteria, Sutterellaceae, and o_Clostridia_vadinBB60_group were the characteristic bacteria, whereas, in GC Piezo1**+/+** mice, FAM, Actinobacteria, Corynebacteriales, Deferribacteraceae, Enterococcaceae, Peptostreptococcaceae, Alphaproteobacteria, Burkholderiales, Bacteria, Rhizobiales, Sghingomonadaceae, Peptococcaceae, Ruminococcaceae, Beijerinckiaceae, Burkholderiaceae, Corynebacteriaceae, Bacillaceae, Streptococcaceae, and Butyricicoccaceae were more characteristic (**Figure 6A**). For MAM, Bacteroidota, Muribaculaceae, Bifidobacteriaceae, Akkermansiaceae, and Clostridiaceae were the characteristic bacteria in GC Piezo1**+/+** mice, whereas in GC Piezo1**-/-** mice, Lactobacillales, f_UCG-010 V, Bacterodiaceae, Mycoplasmataceae, Atopobiaceae, and f_Eubacterium_coprostanoligenes_group were more distinctive (**Figure 6B**). All of them are key bacteria involved in the significant difference between the GC Piezo1^+/+^ and GC Piezo1**-/-** mice.

### Microbial potential functions

We used BugBase to predict potential phenotypes containing mobile elements, Gram-negative, Gram-positive, aerobic, anaerobic, and potentially pathogenic. Among all of the phenotypes, GC Piezo1**-/-** mice tend to have more mobile elements and less anaerobic FAM.

**Figure 7:**
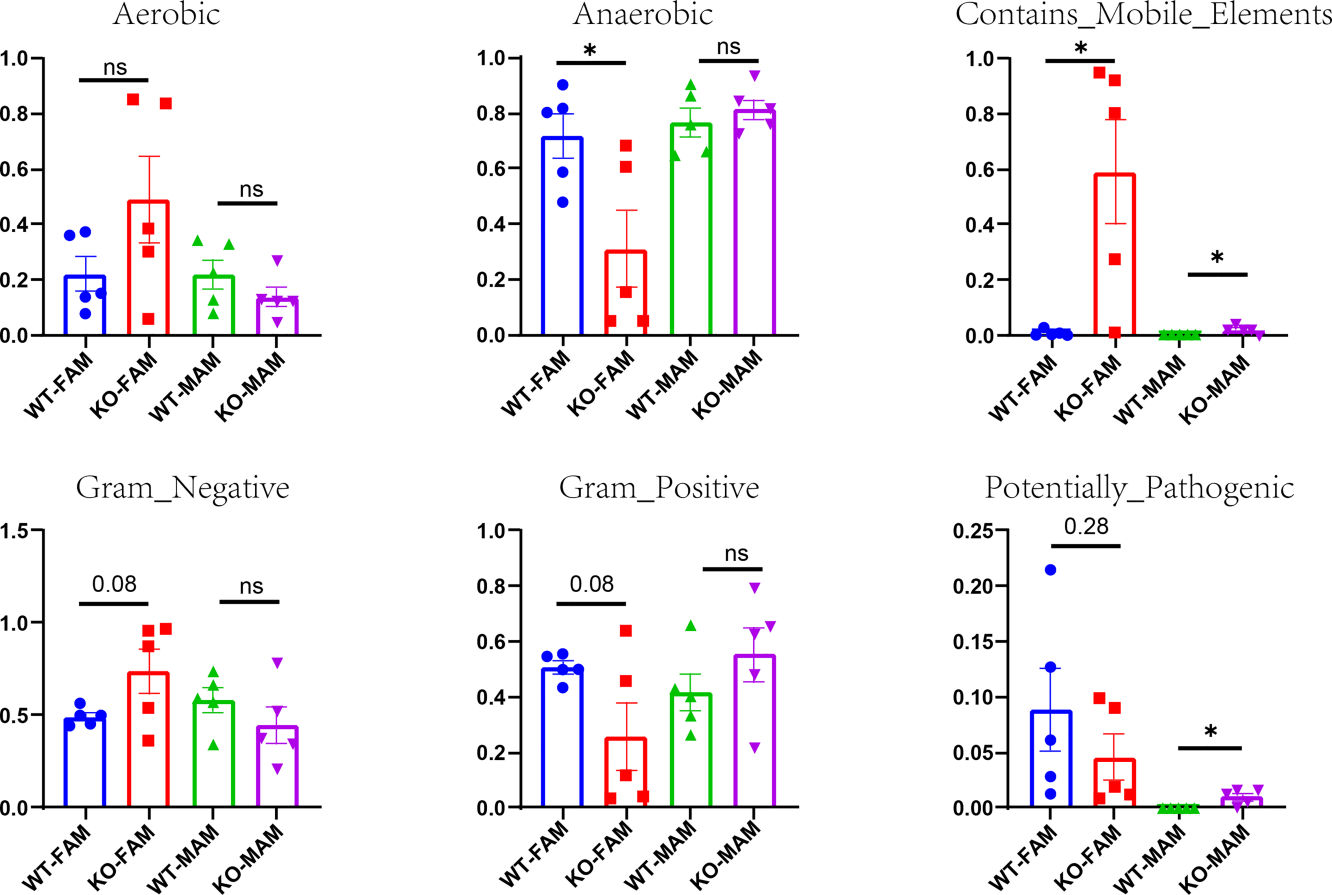
Potential phenotypes (aerobic, anaerobic, containing mobile elements, Gram-negative, Gram-positive, and potentially pathogenic) of two groups in FAM and MAM. *p < 0.05, ns, no significance.

Meanwhile, the abundance of potentially pathogenic and mobile element-containing bacteria was significantly increased in MAM (**Figure 7**), which was mainly due to some reduced taxa that belong to the genus Proteobacteria. These results demonstrated that the protective effects of Piezo1 on GCs were related to the regulation of microbial metabolites.

## Discussion

The canonical function of GCs is to product mucus-associated proteins. Increasing evidence has recently started to reveal the role of GCs in regulating the mucus layer. Muc2 deficiency results in impaired epithelial barrier function, imbalance in gut microbiota, spontaneous colitis, and tumor (Van der Sluis et al., 2006). Loss of TFF3, AGR2 was linked to impaired mucus and increased susceptibility to dextran sodium sulfate-induced colitis (Mashimo et al., 1996; Park et al., 2009)., GC Piezo1^-/-^ mice exhibited decreased expression of Mucin2, TFF3, AGR2, and thinner mucus layer, which was consistent with the decreased GC numbers. In addition, the transcription factor Spdef, which is expressed in the most differentiated GCs and required for proper GC differentiation, maturation, and Mucin2 production (Noah et al., 2010) was decreased in GC Piezo1^-/-^ mice. Spdef^-/-^ mice showed altered mucus phenotype and susceptibility to colitis (Nyström et al., 2021). The increased DOCK4, acts as upstream regulator of Spdef, might be the negative feedback to decreased GC numbers (Qin et al., 2021). The down-regulation of Spdef might be the result of decreased differentiated GC numbers. Furthermore, the slight rise of pro-inflammatory factors like IL-6, CXCL1, and CXCL2 might suggest possible spontaneous colitis in a long observation period. Decreased antimicrobial peptides such as Reg3b and Reg3g, which can be secreted by goblet cells in colon and participate in maintaining intestinal homeostasis (Cash et al., 2006; Xia et al., 2021), also reflected the dysfunction of GC without Piezo1. Therefore, we highlight acritical role of GC Piezo1 in GC function and mucus layer.

Mucus layer acts as the frontline defenders and mediators dictate host-microbe interactions in the intestinal tract during health and disease (Allaire et al., 2018). We discovered that the α-diversity was decreased in FAM but significantly increased in MAM. A lower α-diversity of FAM is always related to inflammatory bowel disease (Pittayanon et al., 2020). Similarly, MAM is considered to be involved in the pathophysiology of IBD because microorganisms directly adhere to epithelial cells (Atarashi et al., 2015). In addition, pathogenic bacteria such as Lactobacillus johnsonii, colibactin-producing Escherichia-Shigella, and mucin-degrading Oscillospiraceae were increased in the MAM, which are involved in promoting an intestinal damage inflammatory response (Raimondi et al., 2021).

Bacterial composition analysis altered in GC Piezo1^-/-^ mice. The ratio of Firmicutes/Bacteroidota (F/B) has been considered an important indicator in the treatment of Obesity and Inflammatory Bowel disease (Stojanov et al., 2020). Bacteroides have been reported to improve inflammation and are involved in the regulation of immune responses (Mazmanian et al., 2008). A previous study showed that the ratio of F/B is higher in obese people than in lean people (Ley et al., 2006; Indiani et al., 2018)., Increased ratio of F/B in GC Piezo1^-/-^ mice responsed to the altered status of gut microbes. In addition, short-chain fatty acid (SCFA)-producing bacteria, such as *Akkermansia muciniphila*, Ruminococcus, and Muribaculaceae, were decreased in GC Piezo1^-/-^ mice (Louis and Flint, 2017; Plovier et al., 2017)., Pathogenic microorganisms such as *Helicobacter hepaticus* and Escherichia-Shigella, which cause diarrhea, were abundant in GC Piezo1-/- mice (Peng et al., 2020; Zhu et al., 2021)., The inner mucus layer in the colon containing feces is much thicker than that in the empty distal colon. Normally, the inner mucus layer devoid of bacteria, confining the bacteria to the feces (Kamphuis et al., 2017). But weakened mucus layer lost its’ function of confining the microbiota to feces and could lead to altered microbiota composition because of deficiency of Piezo1 on GCs. LEfSe analysis also reveals the altered predominance of microbiota in GC Piezo1**-/-** mice. Above all, Piezo1 is required for GCs to maintain the normal function and shape of the composition of microbiota.

GCs are specialized intestinal epithelial cells within the intestinal epithelium and play an essential role in maintaining tissue homeostasis (Allaire et al., 2018). Weakened colonic mucus barrier, which can lead to infiltration of antigens, toxins, and pathogens from the intraluminal environment into the mucosal tissue is an early event in ulcerative colitis pathogenesis (Grootjans et al., 2013; van der Post et al., 2019). GCs can detect and respond to microbial challenge by a coordinated expulsion of whole mucus granules (Johansson and Hansson, 2016b). GC Piezo1^-/-^ mice might not release enough mucus granules and been more sensitive to pathogenic bacteria due to decreased GC numbers. Our study clearly found that GC Piezo1 engaged in regulating mucus layer and spatial reorganization of microbiota. Thus, GC Piezo1**-/-** mice can be used as a new animal model to understand the relationship between intestinal mechanical forces applied to GCs and the microbiota composition.

However, there are some limitations to our study. First, we did not investigate the time course of changes in the gut microbiota and the sensitivity to dextran sodium sulfate-induced colitis in GC Piezo1^-/-^ mice. Second, we failed to evaluate samples of the small intestine because of its fluidity. Third, mechanosensation is important for normal mucosal function and homeostatic microbiota, yet the mechanism by which Piezo1 converts mechanical stimuli and regulates the mucus layer and organization of microbiota needs further study.

## Conclusion

In conclusion, our study demonstrated that GC Piezo1 is involved in orchestrating mucus layer and thus organization of intestinal microbiota, which suggests its importance in intestinal mucus barrier.

## Data availability statement

The data presented in the study are deposited in the NCBI repository, accession number PRJNA874199.

## Ethics statement

The animal study was reviewed and approved by Tongji Medical College Ethics Committee.

## Author contributions

YL performed the major part of experiments, FF and YX helped breeding animals, JW and XL helped with data analyze. GL, TB, and XH provided technical support. JS designed the research, supervised the formal analysis, and reviewed and revised the manuscript. All authors contributed to the article and approved the submitted version.

## Funding

This work was supported by the National Natural Science Foundation of China (Grant nos. 81873553 and 81670488).

## Conflict of interest

The authors declare that the research was conducted in the absence of any commercial or financial relationships that could be construed as a potential conflict of interest.

## Publisher’s note

All claims expressed in this article are solely those of the authors and do not necessarily represent those of their affiliated organizations, or those of the publisher, the editors and the reviewers. Any product that may be evaluated in this article, or claim that may be made by its manufacturer, is not guaranteed or endorsed by the publisher.
